# Differential outcomes of 609 rapid drug desensitization (RDD) for type I hypersensitivity to platins and taxanes

**DOI:** 10.1186/2045-7022-4-S3-P63

**Published:** 2014-07-18

**Authors:** Joana Caiado, Ana Mendes, Elisa Pedro, Manuel Barbosa, Mariana Castells

**Affiliations:** 1 Hospital de Santa Maria, Centro Hospitalar Lisboa Norte, Immunoallergology Department, Portugal; 2 Brigham and Women's Hospital/Harvard Medical School, Division of Rheumatology, Allergy and Clinical Imm, USA

## Background

Hypersensitivity reactions (HR) to chemotherapy agents are increasingly frequent and lead to the discontinuation of potentially curative drugs. Rapid drug desensitization (RDD) is the only strategy that protects from anaphylaxis, while providing first line treatment options. The safety and efficacy of RDD depends on the outcomes of large series of desensitized patients. We present the outcomes data for platins and taxanes from the Desensitization Unit of the Immunoallergology Department in Lisbon's Santa Maria Hospital.

## Methods

A retrospective review between July 2008 and December 2013 was done. All patients with HR to platins and taxanes who were desensitized were included. The Brigham and Women's Hospital's desensitization protocol with 3 bags (1:100, 1:10 and 1:1 of the final target concentration) and 12 consecutive steps was used (5.8 hours total infusion time). Frequency and severity of breakthrough reactions during the desensitization procedures were analyzed.

## Results

135 patients were desensitized to 140 drugs (5 double desensitizations), with a total of 609 desensitizations. The drugs included platins (carboplatin=32; oxaliplatin=40; cisplatin=3) and taxanes (docetaxel=38; paclitaxel=27), with a mean of desensitization per patient of 4.6 for platins and 4.1 for taxanes. During desensitization, 77 (12.6%) HR occurred in 49 patients (mild=59; moderate=13; severe=5) of which 64 (83%) were induced by platins. All breakthrough HR in the taxanes group occurred during the 1st (9-69%) and 2nd (4-31%) desensitizations. Platin-sensitized patients reacted not only during the 1st (27-42%) and 2nd (18-28%), but also on the 3rd (5-7.8%), 4th (5-7.8%) and other (9-14.4%) desensitizations. Most HR occurred on the last step of the protocol (53-69%). Only one patient did not receive the target dose due to recurrent anaphylaxis.

## Conclusions

97% of this large cohort of platin and taxane hypersensitive patients were able to receive their target dose through the 3 bag, 12-steps protocol with mild or no HR, confirming the efficacy and safety of protocol. Platin-sensitized patients react in multiple desensitizations, supporting the need for repeated desensitization at each exposure. Interestingly taxane desensitizations induced fewer reactions, which were limited to the first 2 desensitizations. Future studies are needed to assess the safety of shorter protocols after the first 2 desensitizations to taxanes.

